# Subclinical parameters of arterial stiffness and arteriosclerosis correlate with QRISK3 in systemic lupus erythematosus

**DOI:** 10.1371/journal.pone.0207520

**Published:** 2018-12-05

**Authors:** Mónica Vázquez-Del Mercado, Felipe de J. Perez-Vazquez, Eduardo Gomez-Bañuelos, Efrain Chavarria-Avila, Arcelia Llamas-García, Karla I. Arrona-Rios, Gustavo Ignacio Diaz-Rubio, Sergio Durán-Barragán, Rosa E. Navarro-Hernández, Bethel P. Jordán-Estrada, Natalia Prado-Bachega, Miguel A. A. Gonzalez-Beltran, Carlos Ramos-Becerra, Fernando Grover-Paez, David Cardona-Müller, Ernesto G. Cardona-Muñoz

**Affiliations:** 1 Universidad de Guadalajara, Centro Universitario de Ciencias de la Salud, Instituto de Investigación en Reumatología y del Sistema Músculo Esquelético (IIRSME), Guadalajara, Jalisco, México; 2 Hospital Civil de Guadalajara Dr. Juan I. Menchaca, División de Medicina Interna, Servicio de Reumatología, CONACyT PNPC, Guadalajara, Jalisco, México; 3 Universidad de Guadalajara, Centro Universitario de Ciencias de la Salud, Instituto de Terapéutica Experimental y Clínica (INTEC), Guadalajara, Jalisco, México; Medical University Innsbruck, AUSTRIA

## Abstract

It is well known that cardiovascular diseases (CVD) are a major contributor of death in systemic lupus erythematosus (SLE) as well in other rheumatic illness. In the last decades, there has been a growing development of different methodologies with the purpose of early detection of CVD. Objective: The aim of this study is to correlate the usefulness of subclinical parameters of vascular aging and QRISK 3–2017 score for early detection of CVD in SLE. Methods: Clinical assessment including systemic lupus erythematosus disease activity index (SLEDAI) and systemic lupus international collaborating clinics / american college of rheumatology damage index (SLICC/ACR DI), laboratory measurements, carotid ultrasound examination, carotid intima media thickness (cIMT) measurement, carotid distention and diameter analysis, arterial stiffness measurement measured by tonometry and QRISK 3–2017 were done. All results were analyzed by SPSS 24 software. Results: We observed correlation between QRISK3 and mean cIMT (rs = 0.534, P < 0.001), PWV (rs = 0.474, P < 0.001), cfPWV (rs = 0.569, P < 0.001) and distensibility (rs = -0.420, P = 0.006). Consistent with above, SLE patients in middle and high risk QRISK 3–2017 showed increased arterial stiffness versus low risk group. Conclusions: We encourage to the rheumatology community to assess cardiovascular risk in SLE patients with QRISK 3–2017 risk calculator as an alternative method at the outpatient clinic along a complete cardiovascular evaluation when appropriate.

## Introduction

Systemic lupus erythematosus (SLE) is the prototype of autoimmune rheumatic diseases characterized by the production of autoantibodies against cell components that might cause widespread organ damage. Worldwide cumulated experience and better understanding of SLE are key points for early diagnosis and treatment, resulting in improved life expectancy. Notwithstanding these efforts, infections and inflammatory activity of SLE continue to be the main causes of death during the first years of diagnosis [1]. However, in the last decades, cardiovascular diseases (CVD) have reached the top of the list of causes of death in autoimmune rheumatic diseases, as a direct consequence is now recognized a reduction in SLE life expectancy up to 20 years [2]. The main clinical manifestation of CVD in SLE is accelerated atherosclerosis. Recently, a well conducted clinical study described the accelerated atherosclerosis process in subgroups of SLE patients by mean carotid plaques. The lupus nephritis subgroup compared to matched non-nephritis lupus patients and healthy controls showed twice carotid atherosclerotic plaques [3]. Development of plaque formation by carotid and femoral arterial ultrasound, has been demonstrated in SLE patients independant of the disease activity status [4]. The Hopkins Lupus cohort described a 2.66 fold increased CVD risk [5]. Recently, it has been reported up to 50 times risk for CVD in SLE *vs* non lupus subjects [6]. Subclinical atherosclerosis is one of the goals for early detection of CVD. Since the establishment of CVD as a major contributor of death in SLE and others rheumatic diseases, there has been a growing development of different methodologies with the purpose of early detection of CVD.

Methods for subclinical atherosclerosis using noninvasive tools are able to detect altered function and/or anatomy of vessels [2, 7]. Measurement of carotid intima-media thickness (cIMT) is well recognized as a good predictor of cardiovascular risk (CVR) in various diseases including rheumatoid arthritis (RA). Also, early vascular aging is an important phenomenon documented in several autoimmune rheumatic diseases. Recently, our group has been reported that RA itself is an independent predictor for arterial stiffness evaluated by carotid-femoral pulse wave velocity (cfPWV) [8].

Use of noninvasive methods for CVR evaluation is a trending topic in systemic autoimmune rheumatic diseases (SARDs). Use of dependent and independent operator methods for detection of increase cIMT, altered PWV and arterial distensibility are examples of reliable methods for CVD risk measurement. The use of scores such as Framingham are valuable for estimate CVR in general population but not in rheumatic diseases since this is underestimated. There is controversy about the use of CVR calculators, since caveats existence in their designed, for example do not include the presence of rheumatic diseases as a risk factor but also the fact that many rheumatic patients are younger than 25 years old, when these calculators take into account older population. In this sense, the use of QRISK 3–2017 [9] might be a good score since incorporates the diagnosis of SLE and/or RA as an additive factor for CVR, but still not younger age [10].

Even the extensive literature dealing about CVR in SLE, still is lacking information that compares the usefulness of noninvasive methods and scores to predict CVR. The aim of this study was to evaluate the correlation of PWV, cIMT and arterial distensibility with the QRISK3 score in a selected population of SLE patients.

## Materials and methods

### Patients

We included SLE patients classified according to the Systemic Lupus International Collaborating Clinics (SLICC 2012) criteria, attending the outpatient rheumatology clinic at the OPD Hospital Civil “Juan I. Menchaca”, Guadalalajara, Jalisco, México. Recruitment period comprised from February 2016 to November 2017. To be included in this exploratory study patients had to be>18 years old without known history of CVD. We excluded SLE patients with history of malignancy, hepatic disease, BMI>40, heart arrhythmia and current pregnancy. Control Subjects (CS) were included matched by age and gender.

### Clinical assessment

Each patient was interviewed using a structured questionnaire to obtain demographic and clinical variables, including disease duration and current treatment. Clinical evaluation was performed by a rheumatologist using the Systemic Lupus Erythematosus Disease Activity Index (SLEDAI) [11] and Systemic Lupus International Collaborating Clinics / American College of Rheumatology Damage Index (SLICC/ACR DI) [12].

### Laboratory measurements

Venous blood samples were collected at the moment of clinical assessment. Serum was obtained by centrifugation of whole blood at 2000 rpm for 15 minutes and aliquotes were stored at -70ºC until used. Erythrocyte sedimentation rate (ESR) was measured using Wintrobe´s method. C reactive protein (CRP) was measured by nephelometry. Total cholesterol (TC), triglycerides (TG), high density lipoprotein cholesterol (HDL-c) and low density lipoprotein cholesterol (LDL-c) were measured by standard techniques. Serum complement proteins C3 and C4 were determined by immunoturbidimetry.

### Carotid ultrasound examination

Briefly, carotid ultrasound examination was done by a physician with formal training in vascular screening, using a vascular color doppler ultrasound (MyLabOne, Esaote, Firenze, Italy) coupled with a 4–13 MHz probe SL3323 and an automated guided software [Guided technique radiofrequency-Quality Intima Media Thickness (QIMT) and Quality Arterial Stiffness in real-time (QAS) Esaote, Maastricht, Holland]. Once the subject was in the supine position it was evaluated the right common carotid artery (RCCA), the left common carotid artery (LCCA) and carotid bulb on both sides [13].

### cIMT measurement

Carotid common artery was explored in a longitudinal view with a B Mode Doppler ultrasound. The carotid edges were tracked by radiofrequency (QIMT) at the posterior wall from the leading edge of lumen intima to the leading edge of media adventitia interface. Six cardiac cycles were acquired automatically by the software in order to assess cIMT measurements [14].

### Carotid distention and diameter analysis

These measurements were obtained by an automated software using radiofrequency for tracking the vascular wall and the motion wall through 6 cardiac cycles. A standard deviation (SD) under a cut-off value of 10, SD of diameter measurement under 0.2 mm and SD of distention measurement under 20 μm. Carotid artery distensibility was calculated as:[(systolic diameter–diastolic diameter /diastolic diameter)/(systolic blood pressure–diastolic blood pressure)]. Also, it was calculated automatically the PWV, compliance coefficient (CC), and stiffness index (alpha and beta) [15].

### Arterial stiffness measurement

Arterial stiffness measured through cfPWV was done as described previously. Briefly, cfPWV was measured by tonometry using Pulse Pen device (DiaTecne s.r.l.; Milan, Italy) [8].

### QRISK 3–2017

It was developed based on the QResearch database [16]. We calculated the QRISK 3–2017 risk calculator score available on line and classified individuals as low (<10%), intermediate (10–14.99%) and high risk (≥15%) [9].

### Ethics

The protocol was approved by the Institutional Review Board Committee with the register 0125/16 of Hospital Civil “Dr. Juan I. Menchaca” of the Universidad de Guadalajara. Research was conducted according to Helsinki criteria 2013. All subjects gave their written consent before enrollment.

### Statistical analysis

Variables were assessed for normality using the Kolmogorov-Smirnov test; values are presented as mean ± SD, median with ranges, or percentages (%), as appropriate. Comparisons were made using one-way ANOVA and Kruskall-Wallis, post hoc tests were carried out using the Tukey and Mann-Whitney U as applicable. Chi square, Pearson and Spearman’s correlations coefficients were also calculated, as appropriate. An ANCOVA analysis was carried out to determined predictive variables. All data were analyzed using SPSS 24.0 software (SPSS Inc. Chicago, IL) and GraphPad Prism version 6.00 for Windows (GraphPad Software, La Jolla, CA), considering a two-tailed level of *P* < 0.05 to be statistically significant for analysis.

## Results

We recruited a total of 50 SLE patients and 82 CS matched by age and gender. Demographic and clinical characteristics are shown in Table 1.

**Table 1:** Clinical and demographic characteristics in SLE and control subjects.

Variable	Study group		*P*
	SLE n = 66	CS n = 82	
Age (years)	**33.8 ± 11.8**	**37.6 ± 12.9**	**0.065**
Male / Female (n)	**55 / 12**	**59 / 23**	**0.176**
BMI, kg/m^2^	**28.1 ± 6.1**	**25.6 ± 4.0**	**0.006**
Disease duration (years)	**6.2 ± 5.3**	**——**	**——**
SLEDAI	**5.1 ± 4.6**	**——**	**——**
SLICC	**0.8 ± 1.1**	**——**	**——**
Scholarship			
Basic (n, %)	**28 (42%)**	**——**	**——**
Upper secondary (n, %)	**22 (33%)**	**——**	**——**
Higher (n, %)	**15 (22%)**	**——**	**——**
Alcoholism (n, %)	**0**	**0**	**N.S.**
HT (n, %)	**11 (16%)**	**0**	**<0.001**
DMT2 (n, %)	**4 (6%)**	**0**	**0.038**
Treatment			
Azathioprine (n, %)	**33 (50%)**	**——**	**——**
Chloroquine (n, %)	**45 (68%)**	**——**	**——**
Methotrexate (n, %)	**13 (20%)**	**——**	**——**
Mycophenolate Mofetil (n, %)	**12 (18%)**	**——**	**——**
Corticosteroids (n, %)	**57 (86%)**	**——**	**——**
Corticosteroids (mg, $\bar{x}$ ± SD)	**11.6 ± 13.5**	**——**	**——**
ANAs (n, %)	**64 (97%)**	**——**	**——**
Anti-dsDNA (n, %)	**60 (91%)**	**——**	**——**
Complement			
C3 (mg/dL, $\bar{x}$ ± SD)	**83.7 ± 21.3**	**——**	**——**
C4 (mg/dL, $\bar{x}$ ± SD)	**20.3 ± 11.8**	**——**	**——**
Carotid plaque (n, %)	**0**	**0**	**——**
cIMT mean (μm)	**524 ± 97**	**542 ± 113**	**0.314**
PWV (m/s)	**5.5 ± 1.4**	**5.8 ± 1.3**	**0.167**
cfPWV (m/s)	**7.2 ± 1.3**	**7.0 ± 1.2**	**0.267**
Distensibility, (10^−3^ / kPa)	**32.0 ± 12.2**	**32.1 ± 13.0**	**0.962**

n: number of subjects; %: SLE: Systemic Lupus Erythematosus; CS: Control Subjects; percentage respect to group; SLEDAI: systemic lupus erythematosus disease activity index; SLICC: systemic lupus collaborating clinics; HT: hypertension; DMT2: diabetes mellitus type 2; PWV: pulse wave velocity; cIMT: carotid intima media thickness; cfPWV: carotid-femoral pulse wave velocity; ANAs: antinuclear antibodies; dsDNA: doble stranded DNA; kg: kilograms; m^2^: square meters; $\bar{x}$: mean; SD: standard deviation; m: meters; s: seconds; kPa: kiloPascal; μm: micrometer

### Measurement of cIMT, PWV, distensibility and cfPWV

Since the BMI was difference between SLE and CS, we decided to do an additional analysis considering BMI WHO criteria classification. The cardiovascular parameters were altered in both groups regardless the BMI (supplemental material S1 Table available on line at https://figshare.com/s/9f353067c6ccf852b90e). For this reason, BMI was considered in the ANCOVA analysis.

### QRISK 3–2017 correlated with non invasive methods to evaluate CVR

**Fig 1:**
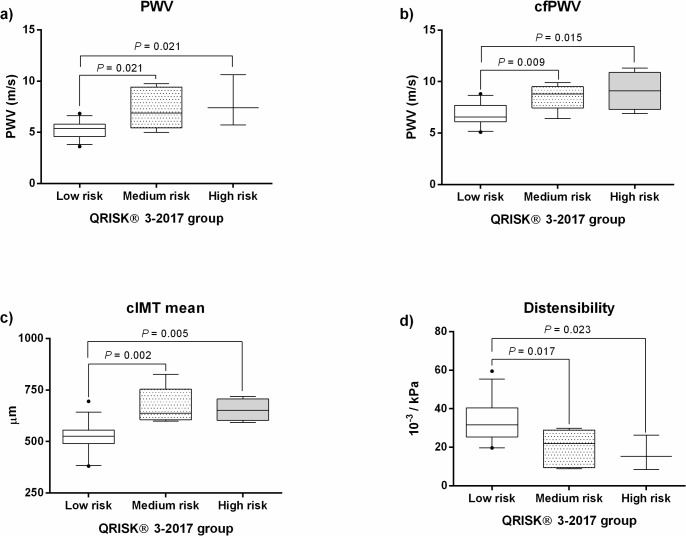
Panel a) Carotid PWV; b) carotid femoral PWV; c) Carotid IMT; d) Carotid Distensibility QRISK 3–2017: based on QResearch database. Error bars indicate percentiles 5 to 95; PWV: pulse wave velocity; m: meters; s: seconds; kPa: kilo Pascal; μm: micrometer; m/s: meters by second.

SLE patients were classified according to the QRISK 3–2017 as low (38 patients, 57.6%), intermediate (5 patients, 7.6%) and high risk (5 cases, 7.6%) with a total of 48 of 66 patients evaluated since the minimum age is 25 years old. Clinical and demographic characteristics in SLE patients by QRISK 3–2017 are showed in Table 2. We observed correlation between QRISK3 and mean cIMT (rs = 0.534, P < 0.001), PWV (rs = 0.474, P < 0.001), cfPWV (rs = 0.569, P < 0.001) and distensibility (rs = -0.420, P = 0.006). Consistent with above, SLE patients in middle and high risk QRISK 3–2017 showed increased arterial stiffness versus low risk group (Fig 1).

**Table 2:** Clinical and demographic characteristics in SLE patients by QRISK 3–2017.

Variable	QRISK 3-2017groups			*P*
	Low risk (<10) (n = 38)	Medium risk (10–14.99) (n = 5)	High risk (≥15) (n = 5)	
Age (years)	**34.6 ± 8.2**	**50.3 ± 11.5**	**44.0 ± 15.2**	0.003*;
Male / Female (n)	**32 / 6**	**4 / 1**	**4 / 1**	**0.951^#^**
BMI, kg/m^2^	**27.6 ± 5.7**	**33.0 ± 7.3**	**33.3 ± 3.2**	0.037**
Disease duration (years)	**5.5 ± 4.3**	**4.9 ± 4.2**	**12.5 ± 7.4**	**0.125**
SLEDAI	**5.8 ± 4.6**	**2.8 ± 1.8**	**4.0 ± 3.2**	**0.344**
SLICC	**0.8 ± 1.2**	**0.8 ± 1.3**	**1.4 ± 1.1**	**0.147**
Scholarship				
Basic (n, %)	**15 (39%)**	**5 (100%)**	**0 (0%)**	**0.063^#^**
Upper secondary (n, %)	**12 (32%)**	**0 (0%)**	**3 (60%)**	
Higher (n, %)	**11 (29%)**	**0 (0%)**	**2 (40%)**	
ESR (n, %)	**16.3 ± 11.5**	**20.3 ± 24.4**	**0.8 ± 1.2**	**0.264**
CRP (n, %)	**7.9 ± 5.1**	**7.0 ± 6.8**	**9.2 ± 6.1**	**0.854**
Treatment				
Azathioprine (n, %)	**19 (50%)**	**2 (40%)**	**3 (60%)**	**0.819^#^**
Chloroquine (n, %)	**29 (76%)**	**2 (40%)**	**2 (40%)**	**0.088^#^**
Methotrexate (n, %)	**11 (29%)**	**2 (40%)**	**0 (0%)**	**0.309^#^**
Mycophenolate Mofetil (n, %)	**7 (18%)**	**0 (0%)**	**1 (20%)**	**0.570^#^**
Corticosteroids (n, %)	**32 (84%)**	**5 (100%)**	**4 (80%)**	**0.711^#^**
Corticosteroids (mg, $\bar{x}$ ± SD)	**9.9 ± 13.3**	**6.9 ± 5.5**	**17.0 ± 20.8**	**0.491^#^**
ANAs (n, %)	**38 (100%)**	**4 (80%)**	**5 (100%)**	**——**
Anti-dsDNA (n, %)	**15 (39%)**	**1 (20%)**	**3 (60%)**	**0.650^#^**
Complement				
C3 (mg/dL, $\bar{x}$ ± SD)	**85.2 ± 15.7**	**69.7 ± 17.1**	**125.0 ± 40.0**	**0.713**
C4 (mg/dL, $\bar{x}$ ± SD)	**18.4 ± 6.0**	**14.7 ± 0.8**	**38.6 ± 13.5**	**0.063**

n: number of subjects; SLE: Systemic Lupus Erythematosus; %: percentage respect to group; SLEDAI: systemic lupus erythematosus disease activity index; SLICC: systemic lupus collaborating clinics; ANAs: antinuclear antibodies; dsDNA: doble stranded DNA; kg: kilograms; m^2^: square meters; $\bar{x}$: mean; SD: standard deviation

*<10 vs 10–14.99 with U Mann-Whitney test as a Kruskal-Wallis’s post-hoc

**<10 vs ≥15 with U Mann-Whitney test as a Kruskal-Wallis’s post-hoc

^#^Fisher exact test

In order to estimate the real impact of each variable (QRISK3 group, age, BMI, etc.) on the cardiovascular parameters measured in this study we design an ANCOVA test (Table 3). QRISK 3–2017 classification remains significant after adjusting by BMI and each level of QRISK 3–2017 impacts an average of fourteen times more than one year on parameters of arterial stiffness.

**Table 3:** Multiple linear regression analysis in SLE patients.

**Model 1, PWV as a dependent variable**		
**Total *R*^2^**	0.623*	
	***β*-Coefficient (95% CI)**	***P***
**Constant**	**2.77 (1.64–3.91)**	**< 0.001**
**QRISK3 group (0 = <10; 1 = 10–14.99, 2 = ≥15)**	**0.91 (0.35–1.47)**	**0.002**
**Age (years)**	**0.07 (0.04–0.10)**	**< 0.001**
**Model 2, cIMT as a dependent variable**		
**Total *R*^2^**	0.495*	
	***β*-Coefficient (95% CI)**	***P***
**Constant**	**407.49 (332.28–482.70)**	**<0.001**
**QRISK3 group (0 = <10; 1 = 10–14.99, 2 = ≥15)**	**56.85 (23.44–90.26)**	**0.001**
**Age (years)**	**3.51 (1.53–5.49)**	**0.001**
Model 3, cfPWV* **as a dependent variable**		
**Total *R*^2^**	0.413*	
	***β*-Coefficient (95% CI)**	***P***
**Constant**	**5.53 (4.28–6.79)**	**<0.001**
**QRISK group (0 = <10; 1 = 10–14.99, 2 = ≥15)**	**0.94 (0.38–1.50)**	**0.002**
**Age (years)**	**0.039 (0.006–0.07)**	**0.023**
**Model 4, Distensibility as a dependent variable**		
**Total *R*^2^**	0.419*	
	***β*-Coefficient (95% CI)**	***P***
**Constant**	**47.23 (36.93–57.51)**	**<0.001**
**QRISK3 group (0 = <10; 1 = 10–14.99, 2 = ≥15)**	**-5.78 (-10.88 –-0.68)**	**0.027**
**Age (years)**	**-0.41 (-0.68 –-0.14)**	**0.004**

*All models were adjusted by BMI; SLE: Systemic Lupus Erythematosus; CI: confidence interval; pulse wave velocity; PWV: pulse wave velocity; cIMT: carotid intima media thickness; cfPWV: carotid-femoral pulse wave velocity

Complement levels, inflammatory markers, disease duration, SLEDAI and SLICC/ACR DI did not correlate with QRISK 3–2017.

## Discussion

There is a gap between the use of methods to evaluate CVR in SLE patients with scores such as QRISK and the usefulness of non-invasive methods. In this scenario, our aim was to evaluate different non-invasive methods for CVR estimation and their correlation with the QRISK 3–2017 risk calculator in SLE patients *vs* CS (Table 1 and Table 2). Our study showed good correlation between QRISK 3–2017 score with non-invasive technology: PWV, distensibility, cIMT and cfPWV (Table 3), although, the methodology for PWV measurement has not been previously validated in SLE population. Extensive literature related to CVR estimation in SLE patients is available, notwithstanding there is no agreement in the results and reliability of methods for CVR estimation. In one study that included 55 SLE and 61 CS matched by age, gender and BMI were evaluated by cfPWV. The cfPWV was increased in CS group but not in SLE patients even though the clinical characteristics of these patients [7]. In addition, there are several SLE reports trying to evaluate different cardiological findings with increased cfPWV [17], such as QT interval length [18], mean blood pressure [19], nocturnal arterial hypertension [20]. When CVR is estimate, the aging is the main influence factor to determine CVR. One study reported increased cfPWV in rheumatoid arthritis (RA), SLE and Behçet´s disease vs CS where aging was an independent risk factor [21]. In contrast, other studies related increased cfPWV with SLE, low C3 complement levels and high CRP [22]. Recently, a meta-analysis of cfPWV studies in SLE patients concluded that cfPWV was increased *vs* CS [23]. In addition, in type 2 diabetes, it was explored the consequences of long term increased arterial stiffness on microvascular damage [24]. A relationship between albuminuria, low glomerular filtration rate, peripheral neuropathy and increased cfPWV was found [25]. Since microvascular damage is prominent in SLE, it would be interesting to conduct a study focus on retinal periphery and/or nailfold capillaroscopy findings associated to measurement of arterial stiffness, in order to give insights into the CVD pathogenesis in SLE population.

In last decades, cIMT measurement has demonstrated to be a good predictor for cardiovascular events in SLE population *vs* CS based mainly on increased cIMT and the presence of plaques [2], also as a tool to look for early atherosclerosis, even the heterogeneity between number of segments, wall characteristics, uni or bilateral evaluation besides dependent or independent operator B mode ultrasound [26–28]. Some reports with less than 30 SLE cases, associated increase cIMT with steroid use, ESR, TG, TC, HDLc, SLEDAI, SLICC, urine protein concentration in 24 hours, ATH and aging [29].

In agreement with our results [30], in this report the results showed non statistical significant differences between PWV, cIMT, distensibility and cfPWV when compared SLE vs CS (Table 1).

Notwithstanding in our study, we were able to showed that cfPWV behaves very similar to carotid measurements of PWV, cIMT and distensibility in SLE patients, meaning that carotid status might be a mirror of the whole aortic arteries.

## Conclusions

Based on our results, we encourage to the rheumatology community to assess cardiovascular risk in SLE patients with QRISK 3–2017 risk calculator as an alternative method at the outpatient clinic besides a complete cardiovascular evaluation.

## Supporting information

S1 Table Age and cardiovascular parameters assessed in SLE and CS respect to BMI. n: number of subjects; CS: control subject; SLE: Systemic Lupus Erythematosus; BMI: Body Mass Index; PWV: pulse wave velocity; cIMT: carotid intima media thickness; cfPWV: carotid-femoral pulse wave velocity; m/s: meters by second; kPa: kilo Pascal; μm: micrometer; kg: kilogram; m^2^: square meter; * U Mann-Whitney test.
Available at: https://figshare.com/s/9f353067c6ccf852b90e.
(DOCX)
