# Supplementary material for: Subclinical parameters of arterial stiffness and arteriosclerosis correlate with QRISK3 in systemic lupus erythematosus
Source: PLoS One. 2018 Dec 5;13(12):e0207520. doi: 10.1371/journal.pone.0207520 (PMC6281193; doi:10.1371/journal.pone.0207520)
Supplement: S1 Table — n: number of subjects; CS: control subject; SLE: Systemic Lupus Erythematosus; BMI: Body Mass Index; PWV: pulse wave velocity; cIMT: carotid intima media thickness; cfPWV: carotid-femoral pulse wave velocity; m/s: meters by second; kPa: kilo Pascal; μm: micrometer; kg: kilogram; m2: square meter; * U Mann-Whitney test. Available at: https://figshare.com/s/9f353067c6ccf852b90e. (DOCX) [file pone.0207520.s001.docx]

**S1 Table. Age and Cardiovascular Parameters Assessed in SLE and CS respect to BMI.**

|  |  |  |  |
| --- | --- | --- | --- |
|  | **SLE** (n= 66) | **CS** (n= 82) | ***P**** |
| **BMI 18.5 – 24.9 kg/m^2^** |  |  |  |
| Age (years) | 29.8 ± 10.3 | 37.6 ± 14.7 | 0.080 |
| cIMT mean (µm) | 482.7 ± 81.5 | 529.6 ± 114.0 | 0.103 |
| PWV (m/s) | 5.1 ± 0.9 | 5.9 ± 1.3 | **0.016** |
| cfPWV (m/s) | 6.8 ± 1.2 | 6.8 ± 1.1 | 0.977 |
| Distensibility, (10^-3^ / kPa) | 36.0 ± 10.3 | 33.8 ± 13.7 | 0.349 |
| **BMI 24.9 – 29.9 kg/m^2^** |  |  |  |
| Age (years) | 33.4 ± 10.6 | 39.1 ± 11.2 | 0.093 |
| cIMT mean (µm) | 510.2 ± 76.4 | 560.5 ± 103.6 | 0.105 |
| PWV (m/s) | 5.3 ± 1.0 | 5.8 ± 1.3 | 0.237 |
| cfPWV (m/s) | 7.0 ± 1.1 | 7.4 ± 1.1 | 0.128 |
| Distensibility, (10^-3^ / kPa) | 37.3 ± 12.6 | 29.5 ± 13.5 | 0.155 |
| **BMI >30 kg/m^2^** |  |  |  |
| Age (years) | 39.0 ± 13.3 | 35.4 ± 10.6 | 0.555 |
| cIMT mean (µm) | 583.2 ± 108.3 | 568.4 ± 130.5 | 0.623 |
| PWV (m/s) | 6.2 ± 2.0 | 5.7 ± 1.0 | 0.541 |
| cfPWV (m/s) | 7.7 ± 1.5 | 6.3 ± 0.8 | **0.048** |
| Distensibility, (10^-3^ / kPa) | 28.4 ± 17.6 | 31.5 ± 6.2 | 0.136 |
|  |  |  |  |

n: number of subjects; CS: control subject; SLE: Systemic Lupus Erythematosus; BMI: Body Mass Index; PWV: pulse wave velocity; cIMT: carotid intima media thickness; cfPWV: carotid-femoral pulse wave velocity; m/s: meters by second; kPa: kilo Pascal; µm: micrometer; kg: kilogram; m^2^: square meter; * U Mann-Whitney test
